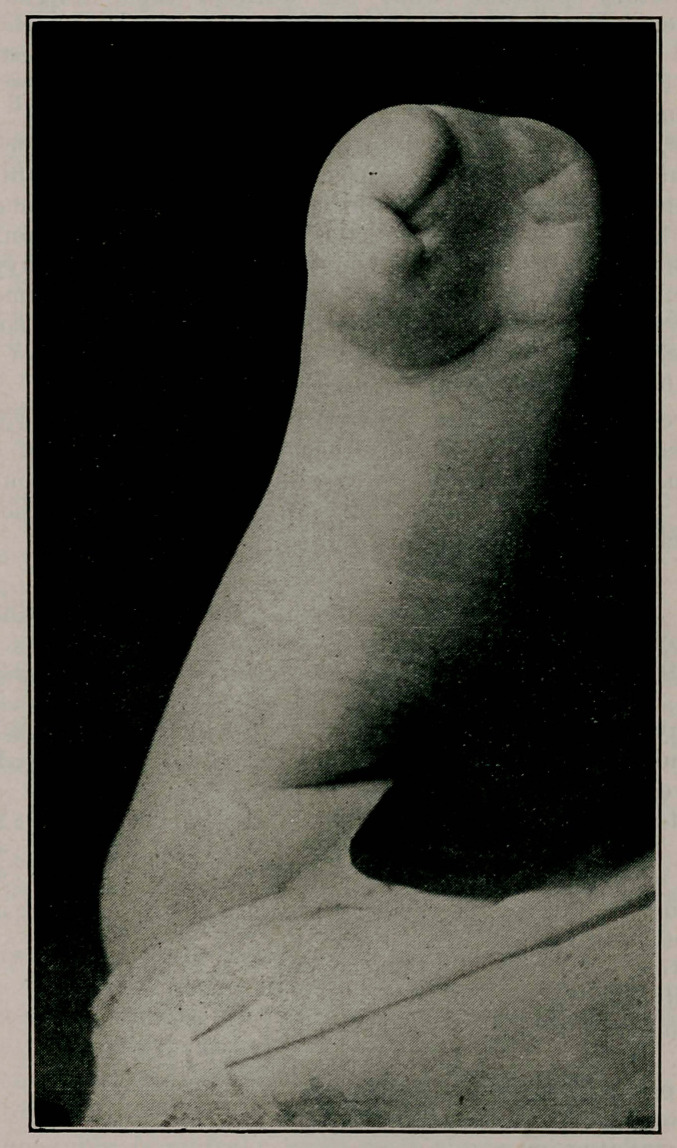# Spontaneous Amputation

**Published:** 1916-10

**Authors:** 


**Spontaneous Amputation.** M. W. Theylis of Wakefield, R. I., Med. World, July reports the following case. (Illustration by courtesy of editor). A woman aged 53, the last of the full blooded Narragansett Indians, had had attacks of diarrhoea for five years, apparently from pancreatic failure. Nov. 1914, she developed general oedema and the left arm became gangrenous, the line of demarcation being established in Feb. 1915, 4 inches above the elbow. In April, she had gained in general health so as to be able to go out doors, the gangrenous fore-arm separating and the bones showing separation of periosteum. Operation had been repeatedly refused. In June, 3% of sugar was found in the urine, which had previously been free from sugar on several examinations, even within a few days. In July, the radius and ulna were sawed off, 5 inches from the elbow, without anaesthetic and in Aug., 2 inches more were sawed off. In Oct. the patient fell and broke off the stump of the radius, from the elbow and in

Nov. the same accident caused the separation of the ulna. Two discharging points were left but these closed in 3 weeks. The ultimate result of the spontaneous amputation is shown in the cut. The patient is now in good general health and able to do practically all the work of her home.

## Figures and Tables

**Figure f1:**